# Selective sensitivity of betel nut-related oral squamous cell carcinoma to evodiamine through cell cycle arrest, growth inhibition and apoptosis induction

**DOI:** 10.7150/jca.136981

**Published:** 2026-07-30

**Authors:** Yin-Che Lu, Kai-Liang Tang, Chih-Hsien Liao, Yi-Zhen Li, Chin-Ho Kuo, Pin-Tzu Chen, Shu-Hsin Chen, Ying-Ray Lee

**Affiliations:** 1 Division of Hematology-Oncology, Ditmanson Medical Foundation Chia-Yi Christian Hospital, Chiayi, Taiwan.; 2 Taichung District Agricultural Research and Extension station, Ministry of Agriculture, Changhua County, Taiwan.; 3 Department of Medical Research, Ditmanson Medical Foundation Chiayi Christian Hospital, Chiayi, Taiwan.; 4 Bone and Joint Research Center, Chang Gung Memorial Hospital, Taoyuan, Taiwan.; 5 School of Medicine, College of Medicine, Kaohsiung Medical University, Kaohsiung, Taiwan.; 6 Department of Microbiology and Immunology, College of Medicine, Kaohsiung Medical University, Kaohsiung, Taiwan.; 7 Master of Science Program in Tropical Medicine, College of Medicine, Kaohsiung Medical University, Kaohsiung, Taiwan.; 8 Faculty of Post-Baccalaureate Medicine, College of Medicine, Kaohsiung Medical University, Kaohsiung, Taiwan.; 9 Center for Tropical Medicine and Infectious Disease, Kaohsiung Medical University, Kaohsiung, Taiwan.; 10 Department of Medical Research, Kaohsiung Medical University Hospital, Kaohsiung, Taiwan.

**Keywords:** evodiamine, anti-cancer, OSCC, apoptosis

## Abstract

Head and neck cancer, particularly oral squamous cell carcinoma (OSCC), remains a major global health burden, with especially high incidence and mortality in Taiwan due to the widespread habit of betel quid chewing. Betel nut mediated OSCC (BN-OSCC) is characterized by aggressive clinical behavior, including increased risks of recurrence, metastasis, and poor survival outcomes, and exhibits limited responsiveness to conventional chemoradiotherapy. Therefore, the development of novel therapeutic agents and radiosensitizers is urgently needed.

Evodiamine (EVO), a bioactive quinolone alkaloid isolated from *Evodia rutaecarpa*, has demonstrated broad anti-tumor activities across multiple cancer types, including inhibition of proliferation, suppression of metastasis, and induction of apoptosis. Although previous studies have reported the anti-OSCC effects of EVO through modulation of pathways such as Akt, EPRS1, and RAGE, its specific impact on BN-OSCC remains unclear.

In this study, we investigated the anti-cancer effects of EVO in BN-OSCC and non-BN-OSCC cell lines. Our results demonstrate that EVO significantly inhibits cell viability and proliferation in a dose-dependent manner in BN-OSCC rather than non-BN-OSCC cells. Importantly, EVO induces apoptosis through activation of the caspase-dependent pathway, as evidenced by increased cleavage of caspase-3, caspase-9, and PARP. Furthermore, EVO treatment leads to mitochondrial dysfunction and enhances pro-apoptotic signaling, suggesting involvement of the intrinsic apoptotic pathway.

Collectively, these findings indicate that EVO exerts potent anti-tumor effects in BN-OSCC cells by triggering caspase-dependent apoptosis. This study provides novel insights into the therapeutic potential of EVO and supports its development as a promising candidate for the treatment of betel nut mediated oral cancer.

## Introduction

Head and neck cancers, including malignancies of the oral cavity, such as the buccal mucosa, gingiva, and tongue, represent a major global health burden. These cancers rank the sixth common malignancy worldwide, with approximately 650,000 new cases and 200,000 deaths annually. In the USA, nearly 54,000 new cases are diagnosed each year, of which more than 99% are histologically classified as squamous cell carcinomas 1. Notably, in Taiwan, head and neck cancers are the fourth leading cause of cancer-related mortality and the sixth most prevalent cancer overall 2, underscoring its significant regional impact.

Although tobacco smoking and alcohol consumption are considered well-established risk factors for oral carcinogenesis 3, epidemiological evidence has shown that betel quid chewing is a predominant and independent etiological factor in Taiwan and across Southeast Asia 4-6. Individuals who chew betel nut exhibit a markedly increased risk of local recurrence, distant metastasis, and developing secondary primary tumors, as well as significantly poorer disease-specific and overall survival compared with non-chewers 4. In Taiwan, the majority of patients with head and neck cancers are male betel quid users, and a substantial proportion presents with an advanced locoregional disease (stages III-IV). The current standard treatment for these patients typically involves multimodal strategies, including chemotherapy combined with radiotherapy (RT). However, despite aggressive treatment, the five-year survival rate remains below 50% because of intrinsic or acquired therapeutic resistance 7, 8. Therefore, identifying effective therapeutic agents and radiosensitizers for betel nut-associated oral squamous cell carcinoma (BN-OSCC) is urgently necessary.

Natural compounds derived from traditional Chinese medicinal herbs have attracted increasing attention as potential anticancer agents and adjuvants that can enhance the efficacy of conventional therapies 9. Evodiamine (EVO), a quinolone alkaloid isolated from *Evodia rutaecarpa* Bentham, has been extensively studied for its broad spectrum of pharmacological activities, including anti-inflammatory, antimicrobial, antiobesity, neuroprotective, and antiviral effects 10-12. Accumulating evidence has shown that EVO possesses potent antitumor properties across a wide range of human malignancies, including cancers of the esophagus, lung, breast, liver, colon, prostate, bladder, pancreas, stomach, ovary, and thyroid, as well as melanoma, glioblastoma, and T-cell lymphoma 9, 13-17. Mechanistically, EVO has been shown to inhibit tumor cell proliferation, induce cell cycle arrest, suppress metastasis, and trigger apoptosis by modulating multiple signaling pathways 13. Furthermore, EVO has been reported to target cancer stem cells, inhibit epithelial-mesenchymal transition (EMT), and overcome drug resistance, thereby highlighting its therapeutic potential 13.

In the context of OSCC, several studies have reported that EVO exerts antitumor effects by inhibiting cell proliferation and inducing apoptosis 18-21. These effects are partly mediated through the modulation of key signaling pathways, including the Akt pathway, glutamyl-prolyl-tRNA synthetase 1 (EPRS1), and the receptor for advanced glycation-end products (RAGE). In addition, EVO has been shown to suppress invasion and angiogenesis in OSCC cells via RAGE-dependent mechanisms 18, 20, 21. Despite these promising findings, the specific effects and underlying mechanism of EVO in BN-OSCC remain unknown.

Therefore, the present study aimed to investigate the antitumor activity of EVO in BN-OSCC cells, with a particular focus on its potential application as a therapeutic agent and radiosensitizer in this high-risk and clinically challenging subtype of oral cancer.

## Materials and Methods

### Cell lines and cell culture

Four human OSCC cell lines, including SAS, OC2, OC3, and OCSL, were employed in this study. The SAS cell line was obtained from the Japanese Collection of Research Bioresources Cell Bank and cultured in Dulbecco's modified Eagle medium (Gibco, Grand Island, NY, USA) containing 10% fetal bovine serum (FBS) and 1% penicillin-streptomycin. The OC2, OC3, and OCSL cell lines, which were originally established from Taiwanese male patients with histories of alcohol consumption, cigarette smoking, and betel quid chewing 6, were maintained in an RPMI-1640 medium supplemented with 10% FBS and 1% penicillin-streptomycin. All cell lines were incubated at 37 °C in a humidified atmosphere containing 5% CO₂.

### Cell viability analysis

Cell viability was assessed using the CCK-8 assay (Enzo Life Sciences, Farmingdale, NY, USA). In brief, 5 × 10³ cells were seeded into a 96-well plate and treated with different concentrations of EVO (0.5-5 μM; Sigma-Aldrich; St. Louis, MO, USA) or a vehicle control (0.01% DMSO) for the indicated durations (24-72 h). Following treatment, a CCK-8 reagent was added and incubated in accordance with the manufacturer's protocol. Absorbance was measured using a microplate reader, and cell viability was expressed relative to the vehicle-treated control group. Each experiment was conducted in triplicate and independently repeated at least three times.

### Colony formation assay

For colony formation analysis, cells were seeded into six-well plates at 1 × 10³ cells per well and maintained under standard culture conditions at 37 °C. Following overnight attachment, the cells were exposed to either a vehicle control medium (0.01% DMSO) or a medium containing EVO for 24 h. After treatment, the culture medium was replaced, and the cells were allowed to grow for an additional 10 days. Colonies were subsequently fixed and stained with a 10% crystal violet solution (Sigma-Aldrich). Colony morphology was documented, and colony number and size were quantified.

### Cell cycle analysis

To determine the effects of EVO on cell cycle progression, cells were treated with a vehicle control or increasing concentrations of EVO for the indicated time periods (24-72 h). After treatment, the cells were harvested and preserved in cold 70% ethanol overnight. The fixed samples were rinsed two times with PBS and stained with a PI solution (Sigma-Aldrich) at room temperature for 30 min under light-protected conditions. Cellular DNA content was quantified by flow cytometry using a FACScan instrument (Becton Dickinson, San Diego, CA, USA), and the proportion of cells in different cell-cycle phases were calculated using ModFit LT 3.3 software. Each experiment was repeated independently on three separate occasions.

### Cellular apoptosis analysis

Apoptosis was quantified by Annexin V-FITC/PI double staining and flow cytometry 14, 22, 23. Cells were treated with EVO or a vehicle control for the indicated concentrations and incubation periods. Afterward, cells were harvested, washed two times with PBS, and resuspended in a binding buffer containing Annexin V-FITC and PI. After incubation for 15 min in the dark at room temperature, fluorescence signals were measured using a FACScan flow cytometer (Becton Dickinson). For each sample, 1 × 10⁵ cells were analyzed, and the percentage of apoptotic cells was calculated. Three independent experiments were conducted to ensure reproducibility.

### Western blotting

The involvement of apoptotic signaling pathways in response to EVO treatment was evaluated using Western blot analysis. The cells exposed to a vehicle control or EVO were harvested, and the total cellular proteins were extracted. Protein samples were resolved by SDS-PAGE and electrotransferred onto PVDF membranes. The membranes were blocked and sequentially incubated with primary and secondary antibodies. The expression levels of apoptosis-associated proteins, including caspase-3 (#9662, Cell Signaling, Danvers, MA, USA), caspase-8 (#9746, Cell Signaling), and caspase-9 (#9502, Cell Signaling), were analyzed, and GAPDH (GTX100118, GeneTex, Hsinchu City, Taiwan) was used as a loading control. Immunoreactive signals were visualized and quantified in accordance with standard Western blotting procedures.

To clarify the role of caspases in EVO-triggered apoptosis, cells were treated with the pan-caspase inhibitor Z-VAD-FMK (BioVision, Mountain View, CA, USA) before EVO administration. Subsequently, the extent of apoptosis was evaluated by Annexin V-FITC and PI co-staining followed by flow cytometric detection. Comparisons between the inhibitor-treated and untreated groups were used to determine the contribution of caspase activation to EVO-induced cell death.

### Statistical analysis

All quantitative data were obtained from at least three independent experiments and are presented as the mean ± SD. Statistical analyses were conducted using GraphPad Prism 8 (GraphPad Software, USA). The differences among experimental groups were assessed by one-way ANOVA with Fisher's LSD post hoc analysis. Statistical significance was defined as a two-tailed *P* value of < 0.05. The levels of significance are indicated as *P* < 0.05, *P* < 0.01, and *P* < 0.001 throughout the study.

## Results

### Evodiamine inhibits the proliferation of human non-BN-OSCC and BN-OSCC cells

**Figure 1:**
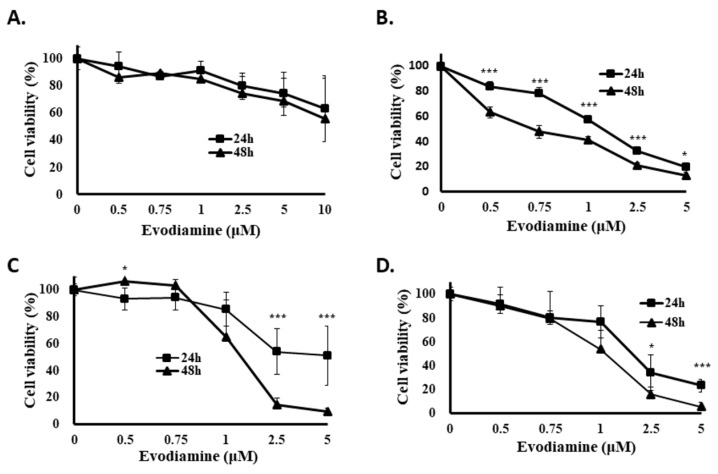
Evodiamine suppresses cell proliferation in human BN-OSCC cells rather than OSCC cells. (A) Human OSCC cell line (SAS), and BN-OSCC cell lines (B: OC2, C: OC3, and D: OCSL) were incubated with DMSO or EVO, and the cell viability was determined with CCK-8 assay. Three independent experiments were conducted.

**Figure 2:**
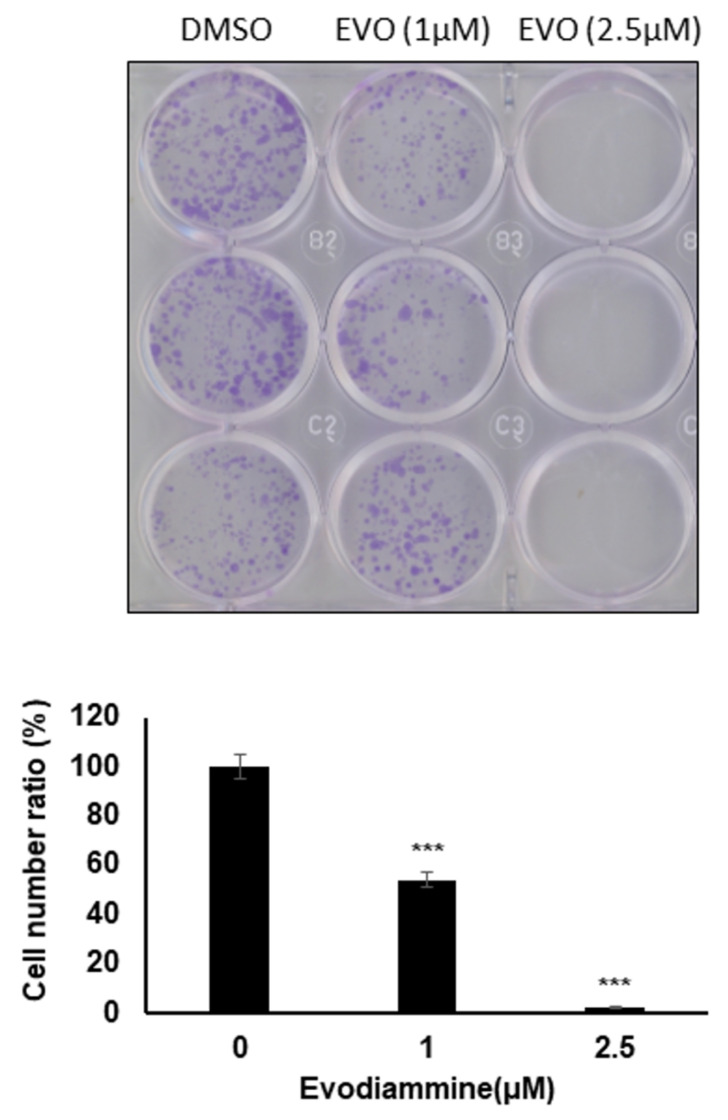
Evodiamine suppresses BN-OSCC cells proliferation. OC2 cells were incubated with evodiamine or DMSO for 24 h, and the colony formation was determined with crystal violet staining after 10days incubation. DMSO was used as negative control. EVO means evodiamine.

Human non-BN-OSCC cell lines, SAS cells, and BN-OSCC cell lines, including OC2, OC3, and OCSL cells, were used to examine the potential effect of EVO. The growth inhibition of OC2, OC3, and OCSL cells was observed in a dosage- and time-dependent manner after EVO treatment (Fig. 1). However, the effect of EVO on growth inhibition resistance in SAS cells was greater than that in BN-OSCC cell lines (Fig. 1). The IC₅₀ value of EVO in SAS cells was not detectable. By contrast, EVO exhibited significant cytotoxicity in BN-OSCC cell lines. In particular, the IC₅₀ values in OC2 cells were 1.45 ± 0.02 μM and 0.71 ± 0.03 μM after 24 and 48 h of treatment, respectively. In OC3 cells, the IC₅₀ values were 2.51 ± 0.16 μM and 1.44 ± 0.10 μM after 24 and 48 h of treatment, respectively. On the contrary, the IC₅₀ values in OCSL cells were 1.94 ± 0.09 μM and 1.15 ± 0.08 μM after 24 and 48 h of treatment, respectively. In addition, the antiproliferative effect of EVO was evaluated in OC2 cells using a colony formation assay, which confirmed that EVO effectively inhibits the proliferation of BN-OSCC cells (Fig. 2). These results indicate that EVO selectively suppresses cell proliferation in human BN-OSCC cells but not in OCSS cells, highlighting its potential application as a therapeutic agent for BN-OSCC.

### Evodiamine induces cell cycle arrest at the G2/M phase in BN-OSCC cells

**Figure 3:**
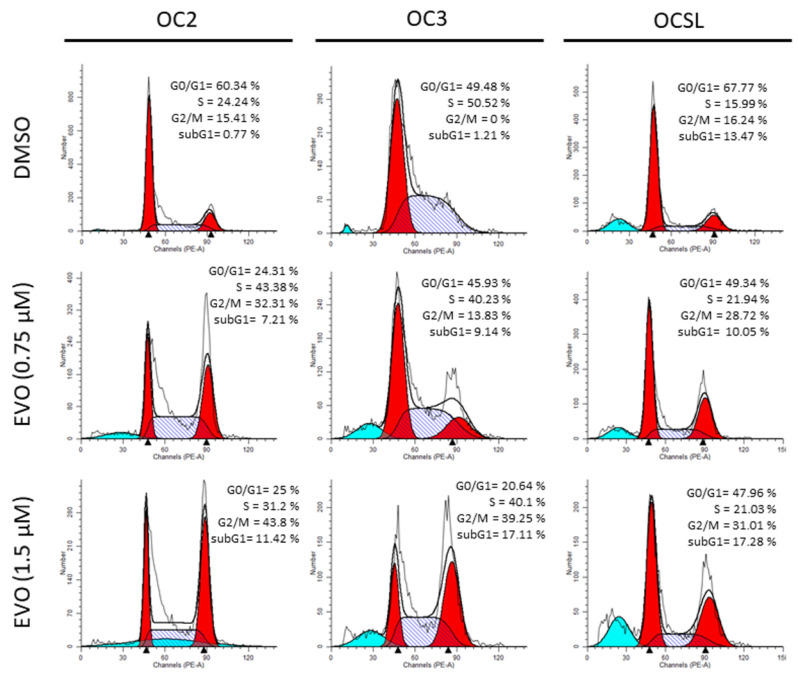
Evodiamine modulates cell cycle in BN-OSCC cells. Human BN-OSCC cell lines (OC2, OC3, and OCSL) were incubated with DMSO or EVO, and the cell cycle was examined with flow cytometry at 12 h post-incubation. DMSO was used as negative control.

Considering that the growth inhibition of BN-OSCC cells under EVO treatment was evident, the cell cycle regulation of EVO treatment in OC2, OC3, and OCSL cells was verified. Figure 3 shows that the cell cycle progression of the abovementioned cells was mostly arrested at the G2/M phase. In addition, EVO slightly increased the subG1 population after 12 h (Fig. 3), indicating that EVO may exert a cytotoxic effect in these cells.

### Evodiamine induces cellular apoptosis in BN-OSCC cells

**Figure 4:**
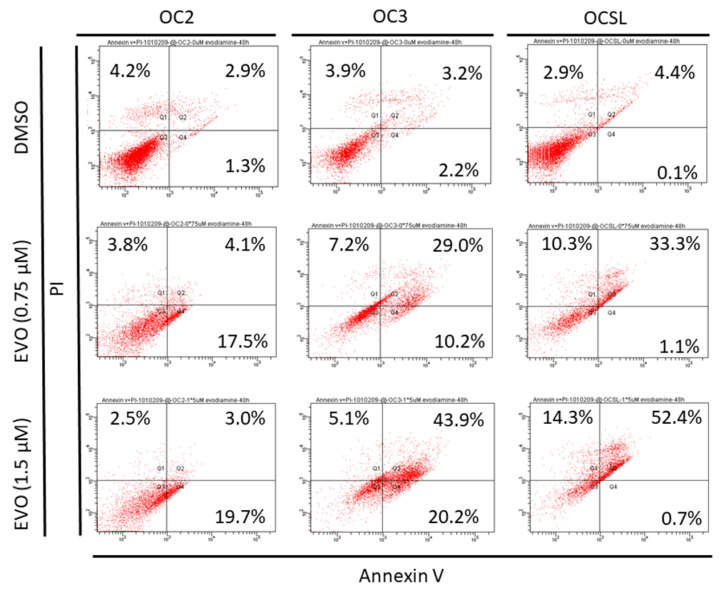
Evodiamine induces cellular apoptosis in human BN-OSCC cells. Human BN-OSCC cell lines (OC2, OC3, and OCSL) were administrated with evodiamine or DMSO for 24 h, and the apoptotic cells were examined with flow cytometry assay. DMSO was used as negative control.

Considering that EVO administration can elevate the sub-G1 group in BN-OSCC cells (Fig. 3), cellular apoptosis induction in these cells was further evaluated using flow cytometry. The result showed that incubation with EVO could significantly elevate cellular apoptosis in BN-OSCC cells (Fig. 4). Moreover, EVO-mediated apoptosis in these cells was OC3 ≥ OCSL > OC2 cells (Fig. 4). These data indicated that EVO treatment induces cell cycle arrest and elevates cellular apoptosis in human BN-OSCC cells.

### Evodiamine induces caspase-dependent apoptosis in BN-OSCC cells

**Figure 5:**
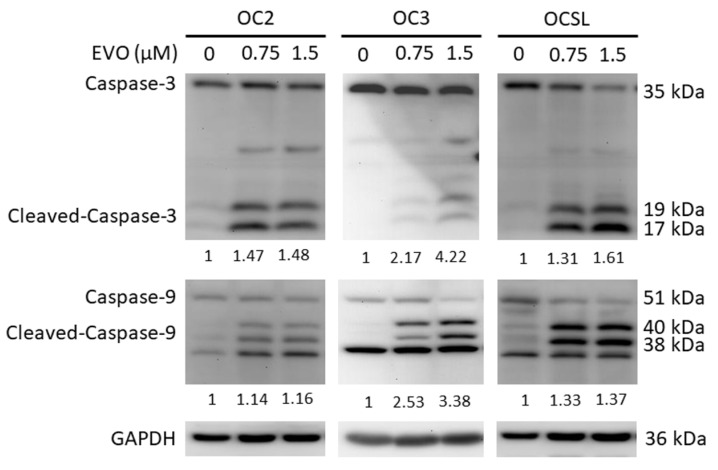
Evodiamine treatment elevates caspase activation in human BN-OSCC cells. Human BN-OSCC cell lines (OC2, OC3, and OCSL) were incubated with evodiamine or DMSO for 24 h, and the activation of caspases were examined with Western blot. DMSO was used as negative control. GAPDH was used as loading control.

EVO induced apoptosis in a dose-dependent manner across all three BN-OSCC cell lines, as demonstrated by Annexin V/PI staining (Fig. 4). To further elucidate the underlying mechanism, the expression and cleavage of key apoptotic markers, including caspase-8, caspase-9, caspase-3, and poly (ADP-ribose) polymerase (PARP), were evaluated. The results showed the significant activation of caspase-9, caspase-3, and PARP following EVO treatment (Fig. 5), whereas caspase-8 remained unchanged. These findings indicate that EVO-induced apoptosis is primarily mediated through the intrinsic (mitochondrial) apoptotic pathway.

**Figure 6:**
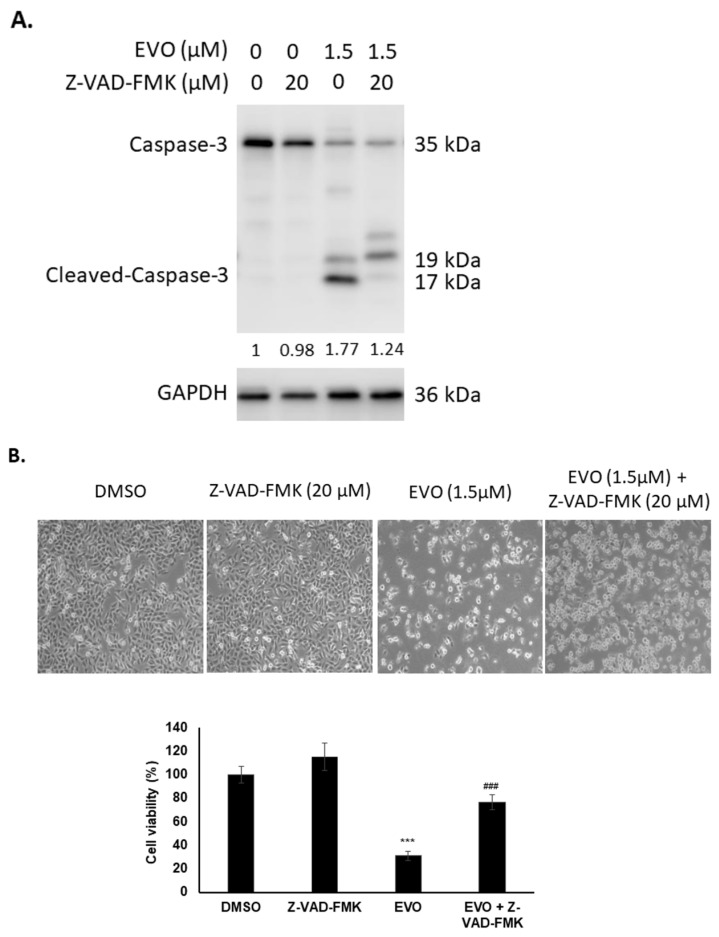
Evodiamine treatment induces caspase-dependent apoptosis in human BN-OSCC cells. Human OC3 cells were incubated with Z-VAD-FMK, evodiamine or combination with Z-VAD-FMK and evodiamine for 24 h, and (A) the activation of caspases was examined with Western blot, and (B) the cell numbers or morphology of OC3 were determined with microscopy. DMSO was used as negative control. Z-VAD-FMK was used to block the activation of caspases. GAPDH was used as loading control. ***: comparing with DMSO group. ###: comparing with EVO group.

To further confirm the involvement of caspase-dependent apoptosis, the pan-caspase inhibitor Z-VAD-FMK was used to block EVO-induced cell death in OC3 cells. As shown in Fig. 6A, co-treatment with EVO and Z-VAD-FMK effectively attenuated caspase activation. Notably, cell viability was significantly restored in the combination treatment compared with EVO treatment alone (Fig. 6B). These results indicate that EVO-induced apoptosis in BN-OSCC cells is caspase-dependent.

## Discussion

OSCC is a heterogeneous malignancy associated with multiple etiological factors, including tobacco, alcohol consumption, and viral infections. By contrast, BN-OSCC is a distinct subtype primarily driven by chronic exposure to areca nut-related carcinogens 24. Betel quid chewing remains a common habit in many regions of the world. Its major alkaloid component, arecoline, has been reported to trigger oxidative stress and genomic alterations, including DNA strand breaks and chromosomal instability, which are considered important events in oral carcinogenesis 25. Compared with conventional OSCC, BN-OSCC exhibits unique biological characteristics, including increased oxidative stress, chronic inflammation, enhanced EMT, and a higher prevalence of genetic alterations, such as TP53 mutations 26-28. Clinically, BN-OSCC is frequently associated with oral submucous fibrosis, which leads to trismus and significantly compromises surgical accessibility and margin clearance 29. These differences contribute to several therapeutic challenges. Although the standard treatment modalities, including surgery, RT, and platinum-based chemotherapy, are similar for OSCC and BN-OSCC 30, patients with BN-OSCC often show reduced sensitivity to chemotherapy and RT, along with a higher risk of local recurrence caused by field cancerization 4, 31-34. Furthermore, the fibrotic and chronically inflamed tumor microenvironment in BN-OSCC may impair drug delivery and modulate immune responses, resulting in variable outcomes with targeted therapy and immunotherapy 32-34. Consequently, BN-OSCC is generally associated with poorer prognosis and greater clinical management difficulties than conventional OSCC. Therefore, the development of effective therapeutic approaches for BN-OSCC is urgently necessary.

EVO has positive effects on various cancers, including thyroid cancer, colorectal cancer, pancreatic cancer, gastric cancer, hepatoma, bladder cancer, tongue squamous cancer, oral cancer, and esophageal cancer 9, 14, 17, 35. Although EVO has been shown to exert anti-OSCC activities, the effect of EVO on BN-OSCC remains unclear. In the present study, EVO exerted potent antitumor effects in human BN-OSCC cells, including the inhibition of cell proliferation, the induction of G2/M cell cycle arrest, and the activation of caspase-dependent apoptosis. Notably, EVO showed selective cytotoxicity toward BN-OSCC cell lines (OC2, OC3, and OCSL), whereas the non-betel nut-associated OSCC cell line (SAS) exhibited marked resistance (Fig. 1), indicating a distinct therapeutic vulnerability in BN-OSCC.

One of the most significant findings of this study is the differential sensitivity between BN-OSCC and non-BN-OSCC cells. The IC₅₀ values of EVO were only detectable in BN-OSCC cell lines, indicating a higher susceptibility to EVO treatment (Fig. 1). This observation is important because BN-OSCC exhibits more aggressive clinical behavior and therapeutic resistance than conventional OSCC 4, 31-34. Given the selective inhibitory effect of EVO, it may target molecular pathways specifically dysregulated in BN-associated carcinogenesis, such as areca nut-induced oxidative stress, DNA damage, and aberrant signaling pathways 15, 32, 34, 36, 37. However, differential responsiveness to EVO was observed among the BN-OSCC cell lines (Fig. 1). Furthermore, the timing and extent of EVO-induced apoptosis varied among the BN-OSCC cell lines (Fig. 4). A similar heterogeneity in drug responsiveness has been reported in oral cancer models. For example, OC2 and OCSL cells exhibit distinct proliferative, migratory, and invasive characteristics, as well as different sensitivities to piperlongumine treatment 38. These findings indicate that the intrinsic biological heterogeneity among BN-OSCC cell lines may contribute to their differential responses to EVO. Nevertheless, the molecular mechanism underlying the relatively low sensitivity of OC2 cells to EVO remains unclear and warrants further investigation.

Mechanistically, our data indicate that EVO induces cell cycle arrest predominantly at the G2/M phase in BN-OSCC cells (Fig. 3). This finding is consistent with previous reports showing that EVO disrupts cell cycle progression by regulating key checkpoint proteins such as cyclin B1, CDK1, and p21 9, 14, 39, 40. G2/M arrest is often associated with DNA damage responses, and it serves as a critical checkpoint to prevent mitotic entry under cellular stress 41. The observed increase in the sub-G1 population further indicates that prolonged cell cycle arrest may lead to apoptotic cell death (Figs. 3 and 4).

In addition to cell cycle arrest, EVO significantly induced apoptosis in BN-OSCC cells, as evidenced by Annexin V/PI staining and increased sub-G1 fraction (Figs. 3 and 4). Among the tested cell lines, OC3 and OCSL cells were more sensitive, followed by OC2 cells (Fig. 4), indicating potential heterogeneity in apoptotic responsiveness within BN-OSCC. Our mechanistic studies revealed that EVO-induced apoptosis is mediated through the intrinsic (mitochondrial) pathway, as demonstrated by the activation of caspase-9, caspase-3, and PARP cleavage, whereas caspase-8 remained unaffected (Fig. 5). Therefore, EVO primarily triggers mitochondrial dysfunction rather than death receptor-mediated apoptosis.

Furthermore, the use of the pan-caspase inhibitor Z-VAD-FMK significantly attenuated EVO-induced caspase activation and restored cell viability, confirming that apoptosis induced by EVO is caspase-dependent (Fig. 6). These findings are consistent with previous studies on other cancer types 9, 42-44, where EVO induces mitochondrial apoptosis by modulating Bcl-2 family proteins, reactive oxygen species generation, and the loss of mitochondrial membrane potential.

From a clinical perspective, the selective antitumor activity of EVO against BN-OSCC cells is particularly noteworthy. Patients with BN-OSCC often have a poorer prognosis and limited therapeutic options because of intrinsic or acquired resistance to conventional chemotherapy and RT. The ability of EVO to selectively target BN-OSCC cells while sparing non-BN-OSCC cells indicates its potential application as a novel therapeutic agent or adjuvant treatment for this specific subtype of oral cancer.

This study has several limitations that should be acknowledged. First, this study was conducted primarily in vitro using established cell lines, which may not fully recapitulate the tumor microenvironment in vivo. Second, the molecular targets underlying the selective sensitivity of BN-OSCC to EVO remain unclear and warrant further investigation. Thus, future studies should identify the key signaling pathways involved and validate these findings in animal models and clinical samples.

In conclusion, our study provides evidence that EVO selectively inhibits proliferation and induces caspase-dependent apoptosis in BN-OSCC cells through G2/M cell cycle arrest and the activation of the intrinsic apoptotic pathway. These findings highlight the potential application of EVO as a promising therapeutic agent for BN-OSCC and support further preclinical and clinical investigations.
